# Chronic unpredictable mild stress promotes atherosclerosis *via* adipose tissue dysfunction in ApoE^-/-^ mice

**DOI:** 10.7717/peerj.16029

**Published:** 2023-09-04

**Authors:** Min Mao, Yalan Deng, Li Wang, Gexin Zhao, Ruomei Qi, Huan Gong, Tao Shen, Yitian Xu, Deping Liu, Beidong Chen

**Affiliations:** 1 The Key Laboratory of Geriatrics, Beijing Institute of Geriatrics, Institute of Geriatric Medicine, Chinese Academy of Medical Sciences, Beijing Hospital/National Center of Gerontology of National Health Commission, Beijing, China; 2 Department of Neurology, Beijing Hospital, Beijing, China; 3 Department of Orthopedic Surgery, David Geffen School of Medicine, University of California, Los Angeles, CA, United States of America; 4 Beijing Union University, Beijing, China; 5 Department of Cardiology, Beijing Hospital, Beijing, China

**Keywords:** Adipose tissue dysfunction, CUMS, Adipocyte hypertrophy, Atherosclerosis, Insulin resistance

## Abstract

**Background:**

Chronic unpredictable mild stress (CUMS) has been shown to exacerbate atherosclerosis, but the underlying mechanism remains unknown. Adipose tissue is an energy storage organ and the largest endocrine organ in the human body, playing a key role in the development of cardiovascular disease. In this research, it was hypothesized that CUMS may exacerbate the development of atherosclerosis by inducing the hypertrophy and dysfunction of white adipocytes.

**Methods:**

The CUMS-induced atherosclerosis model was developed in Western diet-fed apolipoprotein E (ApoE)^-/-^ mice. White adipose tissue (WAT), serum, aortic root, and the brachiocephalic trunk were collected and tested after 12 weeks of CUMS development. The mouse model of CUMS was evaluated for depression-like behavior using the open field test (OFT) and the elevated plus maze (EPM) test. Enzyme-linked immunosorbent assay (ELISA) was conducted to detect serum noradrenaline and urine adrenaline protein levels. Serological assays were used to detect serum low-density lipoprotein (LDL), high-density lipoprotein (HDL), total cholesterol (TC), and free fatty acid (FFA) concentrations. Hematoxylin and eosin (H&E) staining and oil red O were used to detect atherosclerotic plaque area, lipid deposition, and adipocyte size. The mRNA levels of genes related to aberrant adipose tissue function were determined using real-time PCR. Immunofluorescence assay and western blotting were conducted to examine the expression of proteins in the adipose tissue samples.

**Results:**

CUMS aggravated vascular atherosclerotic lesions in ApoE^-/-^ mice. It decreased body weight while increasing the percentage of WAT. The serological results indicated that the concentration of HDL decreased in CUMS mice. Notably, adipocyte hypertrophy increased, whereas the mRNA levels of *Pparg* and its target genes (*Slc2a4* (encodes for GLUT4), *Adipoq*, and *Plin1*) decreased. Further investigation revealed that CUMS increased subcutaneous inguinal WAT (iWAT) lipid synthesis and adipocyte inflammation while decreasing lipid hydrolysis and the expression of HDL-associated protein ApoA-I. Moreover, CUMS aggravated insulin resistance in mice and inhibited the insulin pathway in iWAT.

**Conclusions:**

These findings indicated that CUMS induces adipose tissue dysfunction *via* a mechanism that leads to dyslipidemia, increased inflammation, and insulin resistance in the body, thereby exacerbating atherosclerosis. Notably, CUMS that is involved in decreasing the expression of HDL-associated proteins in adipose tissue may be a crucial link between adipose hypertrophy and advanced atherosclerosis. This study reveals a novel mechanism *via* which CUMS exacerbates atherosclerosis from the novel perspective of abnormal adipose function and identifies a novel potential therapeutic target for this disease.

## Introduction

Globally, cardiovascular disease (CVD) remains the primary cause of mortality. Chronic stress, a common adverse life event, has become an inevitable component of modern life. Chronic stress is harmful to human health and is caused by factors including adversity, depression, anxiety, and loneliness (Dai et al., 2020). Chronic stress-induced depression is widely recognized as a public health issue (Lages et al., 2021). Accumulating evidence indicates that chronic stress, particularly chronic psychological stress, is a primary risk factor for atherosclerotic diseases (Gao & Wang, 2022; Gu et al., 2019; Yao et al., 2019). According to recent research, chronic stress is critically involved in the onset and progression of endothelial injury, dysregulated lipid metabolic diseases, and chronic inflammation (Gao & Wang, 2022). However, the mechanism *via* which chronic stress results in the development of atherosclerosis has not been elucidated yet. Osteoporosis, sarcopenia, and adiposity are all associated inflammatory processes caused by chronic stress (Ilich et al., 2020). However, the regulation of white adipose tissue (WAT) remodeling by chronic stress is unclear.

WAT has been identified as a metabolically active endocrine organ. It regulates numerous body functions, including food intake and energy homeostasis, glucose and lipid metabolism, thermogenesis, neuroendocrine function, immunity, and, most significantly, cardiovascular function (Mattu & Randeva, 2013). Adipose tissue exerts these effects by releasing important chemical mediators called adipokines. Hypertrophic adipocytes secrete various inflammatory adipokines, including interleukin-6 (IL-6), tumor necrosis factor-α (TNF-α), monocyte chemoattractant protein-1 (MCP-1), and serum amyloid A (SAA) (Su & Peng, 2020a). These adipokines cause endothelial dysfunction by increasing vascular reactive oxygen species (ROS) and reducing the bioavailability of nitric oxide (NO) (Akoumianakis & Antoniades, 2019; Mathieu, Lemieux & Després, 2010). Hypertrophic adipocytes also secrete several adipose-derived hormones, such as leptin, resistin, and adiponectin that influence the progression of atherosclerosis. Increased leptin and resistin levels exacerbate inflammation and the accumulation of low-density lipoprotein (LDL) in arteries (Jiang et al., 2014; Katsiki, Mikhailidis & Banach, 2018). Conversely, a decrease in adiponectin aggravates endothelial dysfunction and promotes lipid deposition in macrophages (Zhao, Kusminski & Scherer, 2021). Furthermore, adipose tissue is the most important insulin effector in addition to muscle tissues. Enlarged adipocytes are associated with insulin resistance, decreased glucose uptake, and increased oxidized LDL (Sharma & Singh, 2020; Solis-Herrera et al., 2000; Tokarz & MacDonald, 2018). Adipocytes also play crucial roles in glycolipid metabolism (Ferrero, Rainer & Deplancke, 2020; Pellegrinelli, Carobbio & Vidal-Puig, 2016; Sarjeant & Stephens, 2012), particularly in high-density lipoprotein (HDL) biogenesis, reverse transport, and functional regulation (Zhang et al., 2019, 2010). An abnormal cholesterol metabolism accelerates plaque formation. Therefore, abnormal adipose tissue function represents a significant risk factor for atherosclerosis.

The objective of this research is to examine the effects of CUMS on adipose tissue in atherosclerotic pathology and explore the potential mechanisms underlying the feedback effects of adipose dysfunction on atherosclerosis.

## Materials and Methods

### Materials

Xylene was supplied by Shanghai Macklin Biochemical Co., Ltd. (Shanghai, China). Hematoxylin and eosin (H&E) stains were supplied by Beijing Solarbio Science and Technology Co., Ltd. (Beijing, China). Antibodies against acetyl coenzyme A carboxylase (ACC), fatty acid synthase (FASN), glucose transporter member 4 (GLUT4), and β-actin were acquired from ABclonal Technology Co., Ltd. (Wuhan, China). Antibodies against hormone-sensitive lipase (HSL), phosphorylated HSL (p-HSL), protein kinase B (AKT), and phosphorylated AKT (p-AKT) were acquired from Cell Signaling Technology (Danvers, MA, USA). The antibody against adipose triglyceride lipase (ATGL) and horseradish peroxidase-conjugated goat anti-rabbit and mouse immunoglobulin G secondary antibodies (Mouse-HRP & Goat Anti-Rabbit) were acquired from Abmart Shanghai Co., Ltd. (Shanghai, China).

### Animals

Sixteen ApoE knockout (ApoE^-/-^) male mice weighing 22.5 ± 1.4 g and 16 weeks old were acquired from Beijing HFK Bioscience Co., Ltd. (Beijing, China). All mice were kept individually in a standardized animal room (12 h/12 h light/dark cycle; 22 ± 2 °C; 50–60% relative humidity) and were provided proper diet. Mice were acclimatized for two weeks before initiating the stress procedures. The animals were subjected to all stressors in a room separate from their housing area. For animal experiments, the ARRIVE guidelines, The Animal Act of 1986 (Scientific Procedures), the U.K, and EU Directive 2010/63/EU were followed during the procedures. The Peking University Animal Experimentation Ethics Committee approved the protocols for the animal experiment (permit no. LA2022278).

### CUMS protocol

The male mice were classified into two groups at random (eight mice each): the ApoE^-/-^ CUMS group (fed the Western diet with 12 weeks of chronic unpredictable mild stress) and the ApoE^-/-^ control group (fed the Western diet without any stress). A random number generator was used to generate random seed number for grouping. The Western diet (WD) was administered to mice in both groups (0.15% cholesterol, 21% fat; Beijing HFK Bioscience Co., Ltd., Beijing, China). The CUMS procedures were done as previously described, with slight modifications (Komoto et al., 2022). Each week, ApoE^-/-^ mice were randomly exposed to the following stressors to prevent them from adapting to the CUMS procedure: The tail was clamped and shaken for 1 min; the cage was shaken 15 times (5 s duration each, 10 s apart); the light/dark cycle was reversed (sustain illumination overnight for 12 h, sustain daytime darkness for 24 h, sustain illumination overnight for 12 h again); the mice were placed in cages with the odor of other mice for 1 h twice weekly; the mice were exposed to intermittent noise (70 dB) for two cycles. Then, the mice were exposed to three cycles of low-intensity strobe light (150 flashes per min). The mice were restrained in a plastic tube for 2 h without food and water. The cage was placed on a platform at a 45° angle and rotated the cage 180° per h, and the bedding was wet for 24 h. The control mice were kept in a separate room, were not exposed to these stressors, and were not allowed to get into contact with the stressed mice. Body weight, urine epinephrine concentrations, serum biochemical indicators, and atherosclerotic plaque size were measured after 12 weeks of CUMS. To ensure the unpredictability of the occurrence of stimulation, these stressors were changed randomly as one stressor per day in the mice and administered at the same time each day. After CUMS model establishment and behavioral assessment, the mice were kept unconscious by intraperitoneal injection of 1% sodium pentobarbital 0.1 ml/20 g for euthanasia.

### Open field test

OFT was conducted to assess exploratory and autonomous behavior and tension of experimental animals in new environments. A higher anxiety level is correlated with less independent activity in OFT (de Souza et al., 2015). Nanjing Calvin Biotechnology Co., Ltd. (Nanjing, China) provided the open field device (50 cm × 50 cm × 40 cm). There were 16 cells in the upper reaction chamber, of which the peripheral 12 cells were reported as the peripheral region and the middle four cells as the central region. Experiments were performed in a dimly lit test room with a relatively quiet environment. Mice were kept individually in the central square and allowed to roam freely for 5 min. The total mobility distance and speed of mice in various regions were recorded. An OFT was conducted in a soundless room without human intervention. After each mouse was tested, the reaction chamber was disinfected with a 5% water-ethanol solution to remove any bias caused by the odor of the previous mice.

### Elevated plus maze (EPM) test

As mentioned, the EPM was used to identify anxiety-like behavior (Fan & Tian, 2018). For 5 min, a camera captured the behavior of the mice in the middle of the EPM apparatus. An indication of anxiety-like behavior was the percentage of time spent on open arms. After each test, the maze was thoroughly cleaned.

### Noradrenaline, adrenaline and serum lipid measurement

Blood samples were taken by cardiac aspiration after the mice were anesthetized with 2% isoflurane. Blood was collected in tubes and centrifuged at 3,000 rpm for 15 min at 4 °C by a refrigerated centrifuge (HEMA). Serum noradrenaline (NE) and urine adrenaline protein levels were assayed using ELISA kits (Catalog number: E-EL-0047c and ml002049) from Elabscience Biotechnology Co., Ltd. (Wuhan, China) and Shanghai Enzyme-linked Biotechnology Co., Ltd. (Shanghai, China) according to the provided guidelines. Serum concentrations of LDL, HDL, total cholesterol (TC), and free fatty acid (FFA) were measured by enzymatic method using AU5400 Chemistry System from Beckman Coulter (Brea, CA, USA).

### Tissues collection

After 12 weeks of CUMS treatment, mice were fasted for 12 h with free access to water. Subsequently, mice were anesthetized and euthanized. The brachiocephalic trunks and the hearts containing aortic root were embedded in the optimal cutting temperature compound (OCT) (Sakura Finetek USA Inc., Torrance, CA, USA) and frozen immediately in liquid nitrogen for further analysis. The skin was carefully removed for subcutaneous adipose tissue collection, and adipose tissue was dissected from the inguinal region. For visceral adipose tissue collection, the peritoneum was opened and adipose tissue was dissected from the epididymal region. The margins for dissection were determined based on their anatomical landmarks and standard procedures. The collected adipose tissue was fixed with 4% paraformaldehyde overnight and then transferred to 75% alcohol until paraffin embedding.

### Assessment of atherosclerotic lesions

Frozen sections of 7 µm thickness were prepared from brachiocephalic trunks and the aortic roots to examine atherosclerotic lesions. Hematoxylin and eosin (H&E) or oil red O was used to stain the sections. Data were collected as previously described (Zhang et al., 2022). Specifically, HE staining was manually measured using ImageJ software, and the ratio of lesion area to lumen area was obtained. For oil red O, the lesion area with positive staining was analyzed. The percentage of the stained area was obtained by calculating the oil red O positive area *vs* the area of the aorta cross section multiplied by 100%.

### Analysis of adipocyte size

Explants of subcutaneous and visceral adipose tissue were fixed in paraformaldehyde overnight. The tissues were then dehydrated using an alcohol gradient, embedded in paraffin, sliced into sections of 5 μm thickness, placed on charged glass slides, and subjected to heat treatment. The paraffin-embedded sections (5 μm) were stained with H&E to assess cell size in adipose tissue by counting the number of cells per field of view. Slides were imaged on a Keyence BZ-X800 microscope in 200× visual fields (eight visual fields per group). The cells were counted using AdipoCount software.

### Immunohistochemistry assay

The expression of GLUT4 on the cell membrane was detected by immunohistochemistry. Paraffin-embedded (5 µm) sections of tissues were placed on poly-L-lysine-coated slides, and each case included a negative control. The slides were dried at 60 °C for 1 h. After deparaffinization and blocking of endogenous peroxidase, sections were heated in phosphate-buffered saline (PBS; pH 7.2) at 94 °C for 10 min and then cooled to 25 °C. Following overnight incubation with GLUT4 primary antibody (ABclonal, Woburn, USA) at 4 °C, sections were rinsed three times with PBS, incubated with secondary antibodies (Goat Anti-Rabbit and Mouse-HRP; Abmart, Shanghai, China) at room temperature for 1 h, and then rinsed with PBS. Afterward, 3, 3′-diaminobenzidine tetrahydrochloride (DAB) was utilized to illuminate positive cell staining. The sections were counterstained with hematoxylin. Semi-quantitative image analysis was performed with ImageJ software, and the IHC profiler plugin developed by Varghese et al. (2014) was utilized for this purpose. This software offers a four-tier system providing quantitative percentages for the analyzed data. A scoring system was implemented, with assignments made as follows: high positive (3+), positive (2+), low positive (1+), and negative (0). The simple algebraic formula calculated the final score: Score = 3 × high positive % + 2 × positive % + 1 × low positive %. The data were presented as relative positive IHC score compared to the Con group.

### Real-time quantitative reverse transcriptase polymerase chain reaction (qRT-PCR)

Total RNA was extracted from 100 mg of adipose tissue from each mouse using RNAiso Plus (TaKaRa, Catalog number: 9109). Subsequently, the TaKaRa PrimeScript RT reagent kit (TaKaRa, Catalog number: RR036A) was utilized to reverse transcribe 2 μg of RNA into cDNA. The TaKaRa TB Green Premix Ex Taq II kit (TaKaRa, Catalog number: RR820A) was utilized for conducting qRT-PCR. The reaction conditions, as stated in the reagent manual, were as follows: 95 °C for 30 s, 95 °C for 5 s, and 60 °C for 30 s (40 cycles). The primers are listed in Table 1.

**Table 1:** Primers used for qRT-PCR.

Gene	Sequence	
18*S*	F:	5′-GGAAGGGCACCACCAGGAGT-3′
	R:	5′-TGCAGCCCCGGACATCTAAG-3′
*Pparg*	F:	5′-ACCACTCGCATTCCTTTGAC-3′
	R:	5′-CCACAGACTCGGCACTCAAT-3′
*Slc2a4*	F:	5′-TCCTTCTATTTGCCGTCCTC-3′
	R:	5′-TCAAGTTCTGTACTGGGTTTCAC-3′
*Adipoq*	F:	5′-CTCCTGCTTTGGTCCCTC-3′
	R:	5′-GCCTGGTCCACATTCTTT-3′
*Plin1*	F:	5′-CTGTGTGCAATGCCTATGAGA-3′
	R:	5′-CTGGAGGGTATTGAAGAGCCG-3′
*Acc*	F:	5′-TGAGGAGGACCGCATTTATC-3′
	R:	5′-CTGATGATCGCACGAACAAA-3′
*Fasn*	F:	5′-GCTGGCATTCGTGATGGAGTCGT-3′
	R:	5′-AGGCCACCAGTGATGATGTAACTCT-3′
*Atgl*	F:	5′-ACACCAGCATCCAGTTCAAC-3′
	R:	5′-AAAGGGTTGGGTTGGTTCAG-3′
*Hsl*	F:	5′-TTCTCCAAAGCACCTAGCCAA-3′
	R:	5′-TGTGGAAAACTAAGGGCTTGTTG-3′
*Saa3*	F:	5′-AGAGAGGCTGTTCAGAAGTTCA-3′
	R:	5′-AGCAGGTCGGAAGTGGTTG-3′
*Saa1/2*	F:	5′-AGACAAATACTTCCATGCTCGG-3′
	R:	5′-CATCACTGATTTTCTCAGCAGC-3′
*Il6*	F:	5′-GGATGCTACCAAACTGGATA-3′
	R:	5′-CTCTGGCTTTGTCTTTCTTG-3′
*Tnf*	F:	5′-CGTCGTAGCAAACCACCAAG-3′
	R:	5′-GTCCCTTGAAGAGAACCTGG-3′

### Western blot analysis

RIPA buffer containing protease and phosphatase inhibitors was used to homogenize the adipose tissue (Zhongshan Jinqiao), and the homogenates were centrifuged for 15 min at 4 °C at a rate of 12,000 rotations per min. After being collected, the supernatant was mixed with 5× loading buffer (Beyotime Biotechnology Co., Ltd., Nanjing, Jiangsu, China). Sodium dodecyl sulfate-polyacrylamide gel electrophoresis was utilized to separate samples having equal amounts of protein and transferred to Immobilon®-PSQ polyvinylidene fluoride (PVDF) membranes (Merck Millipore Ltd., Tullagreen Carrigtwohill, County Cork, Ireland). The membranes were incubated with the following primary antibodies for the whole night at 4 °C after being blocked with 5% skim milk: β-actin, total AKT, FASN, ACC, GLUT4, and p-Akt (S473) (1:1,000 dilutions). The PVDF membranes were then incubated for 2 h at room temperature with Goat Anti-Rabbit Mouse IgG-HRP secondary antibodies (1:10,000 dilutions). The membranes were then visualized using the electro-chemiluminescent solution. ImageJ software was used for semi-quantitative analysis. The information of antibodies is listed in Table 2.

**Table 2:** Information of antibodies.

Antibody	Catalog number	Origin	Phosphorylation site
GLUT4	A7637	ABclonal Technology (Wuhan, China)	
ACC	A19627	ABclonal Technology (Wuhan, China)	
FASN	A0461	ABclonal Technology (Wuhan, China)	
ATGL	M30109XS	Abmart Biotechnology Co., Ltd. (Shanghai, China)	
Phospho-HSL	40803S	Cell Signaling Technology Inc. (Massachusetts, United States)	Ser563
HSL	4107S	Cell Signaling Technology Inc. (Massachusetts, United States)	
ABCA1	NB400-105	Novus biologicals (Centennial, United States)	
ABCG1	ab52617	Abcam Plc (Cambridge, United Kingdom)	
APOA1	A14211	ABclonal Technology (Wuhan, China)	
Phospho-AKT	4060T	Cell signaling Technology Inc. (Massachusetts, United States)	Ser473
AKT	E2912	Santa Cruz Biotechnology (California, United States)	
β-actin F4/80 TNF-α IL-6	AC026 GB113373 GB11188 GB11117	ABclonal Technology (Wuhan, China) Servicebio (Wuhan, China) Servicebio (Wuhan, China) Servicebio (Wuhan, China)	
Goat anti-rabbit mouse IgG-HRP	M21003	Abmart Biotechnology Co., Ltd. (Shanghai, China)	

### Intraperitoneal glucose tolerance test (IPGTT)

After a 16-h fast, glucose (2 g/kg) was administered intraperitoneally to the mice. Levels of glucose were examined using blood glucose strips (Jiangsu Yuyue Medical Equipment & Supply Co., Ltd., Danyang, Jiangsu, China) at 0, 15, 30, 60, 90, and 120 min.

### Assessment of insulin sensitivity

Insulin sensitivity was assessed by the insulin tolerance test (ITT). After a 6-h fast, mice were injected intraperitoneally with insulin (0.5 U/kg). Blood glucose levels were measured at 0, 15, 30, 60, 90, and 120 min using blood glucose strips. The constant for plasma glucose disappearance (KITT), which serves as an indicator of peripheral response to insulin, was obtained by a linear regression analysis of plasma glucose concentrations change between 0 and 15 min post-insulin injection, during which the glucose concentration decreased linearly.

### Immunofluorescence assay

Conforming to the standard protocol, immunofluorescence staining was executed using the following antibodies and dilutions: TNF-α (1:500; Servicebio, Wuhan, Hubei, China), IL-6 (1:500; Servicebio, Wuhan, Hubei, China), and F4/80 (1: 1,000; Servicebio, Wuhan, Hubei, China). After being deparaffinized and rehydrated, the samples were subjected to epitope retrieval by microwaving the slides in an EDTA antigen retrieval buffer with a pH value of 8.0 (Servicebio, Wuhan, Hubei, China). The samples were first permeabilized with BioDewax and Clear Solution (Servicebio, Wuhan, China) and blocked with 3% BSA. Only one antigen was found in each stage, which included primary and secondary antibody incubation, as well as tyramine signal amplification (TSA) visualization. After protein blocking and epitope retrieval as mentioned prior, the next antibody was labeled. To finally stain the nuclei of the cells, the samples were counterstained with 4′,6-diamidino-2-phenylindole (DAPI). The immunofluorescent images were visualized and captured using the PANNORAMIC MIDI (3DHISTECH™, Budapest, Hungary) at 200× magnification. Semi-quantitative image analysis was performed with ImageJ software. The data were presented as relative fluorescence intensity to the Con group.

### Statistical analysis

The software SPSS Statistics 25 was used to analyze the data. Normality and homogeneity tests were performed to ensure that the data fulfilled the assumptions for parametric statistics. After examination, it was found that all of the data were normally distributed with equal variances. The Student’s t-test was utilized to compare the means between the two groups. The findings are presented as mean ± SD and were evaluated using independent *t*-tests. The value of statistical significance was fixed at *p* < 0.05.

## Results

### CUMS aggravated vascular atherosclerotic lesions and decreased serum HDL levels

**Figure 1:**
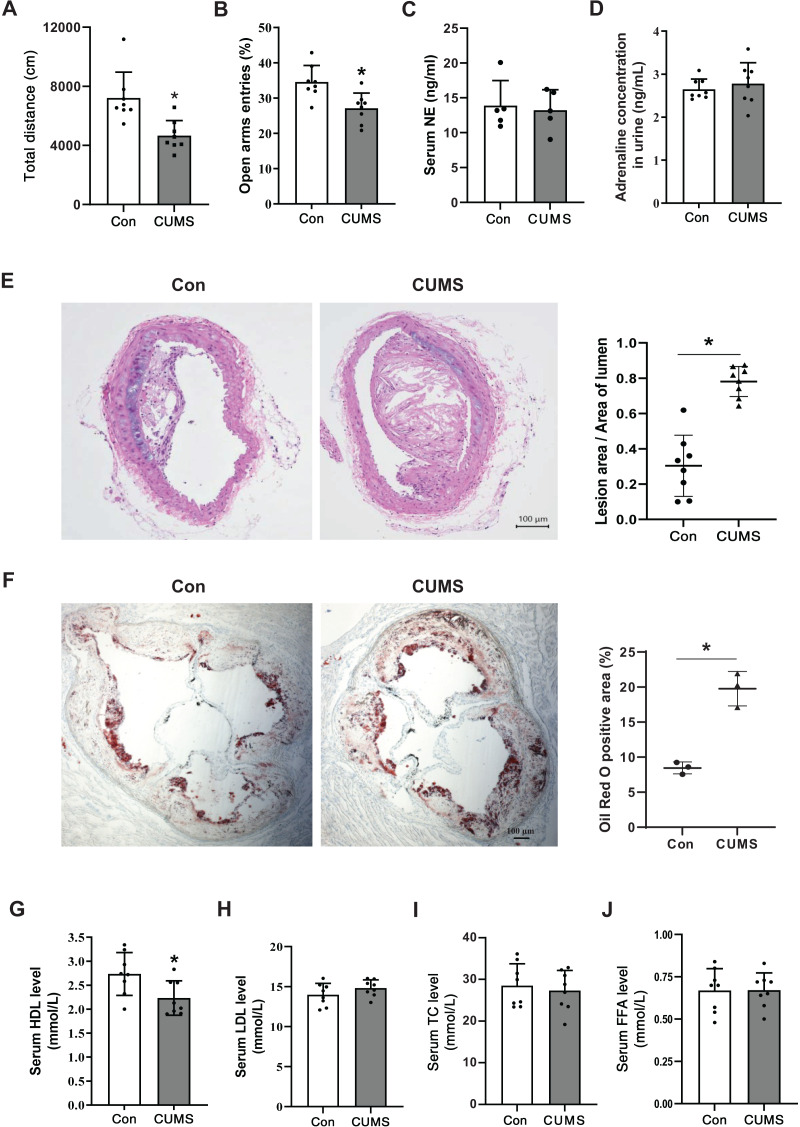
CUMS aggravates vascular atherosclerotic lesions and decreases serum HDL levels. (A) Total locomotive distance of mice in the OFT reaction box; (B) the percentage of entries into open arms relative to the total number of entries into all arms in the EPM; (C) serum NE levels in the Con and CUMS groups measured by ELISA; (D) adrenaline concentrations in mice urine measured by ELISA; (E) representative H&E staining images of the brachiocephalic trunks in the Con and CUMS groups, 100× magnification (Left) and quantitative analysis of lesion area per lumen area (Right); (F) representative images of Oil Red O staining of aortic root sections in the Con and CUMS groups, 40× magnification(Left) and quantitative analysis of the oil red O positive area (Right); (G) serum HDL, (H) LDL, (I)TC and (J) FFA levels in the Con and CUMS groups. The data are expressed as mean ± SD. *p < 0.05, CUMS *vs* Con. *n* = 3–8 per group.

To determine if the CUMS paradigm induced depressive-like behavior, OFT and EPM were executed on a Western diet (WD)-fed ApoE^-/-^ mice (Con) and the WD ApoE^-/-^ mice treated with CUMS for 12 weeks (CUMS). The overall locomotive distance in the OFT reaction chamber and the percentage of entry into open arms were lower in the CUMS group than in the Con group, showing that the CUMS procedure triggered depressive-like behavior (Figs. 1A and 1B). Catecholamines are crucial stress response mediators and have a direct stimulatory effect on adipose tissue lipolysis. Here, the serum NE and urine adrenaline levels were measured in Con and CUMS mice. Results showed that catecholamines levels did not considerably vary between the CUMS and Con groups (Figs. 1C and 1D).

CUMS has previously been shown to aid in the development of atherosclerosis in ApoE^-/-^ mice (Gu et al., 2019). The research findings revealed that CUMS had a significant aggravating effect on the atherosclerotic lesions in both the brachiocephalic trunks and the aortic root of WD ApoE^-/-^ mice when compared to the Con group (Figs. 1E and 1F). Additionally, CUMS was found to significantly decrease serum HDL levels (Fig. 1G). Previously it was shown that elevated basal lipolysis in obese patients is one of the causes of dyslipidemia and atherosclerosis (Li & Sun, 2018). Conversely, the resulting data of this research showed that serum FFA levels were not remarkably elevated in CUMS mice (Fig. 1J).

### CUMS increased body fat percentage and enlarged iWAT adipocytes

**Figure 2:**
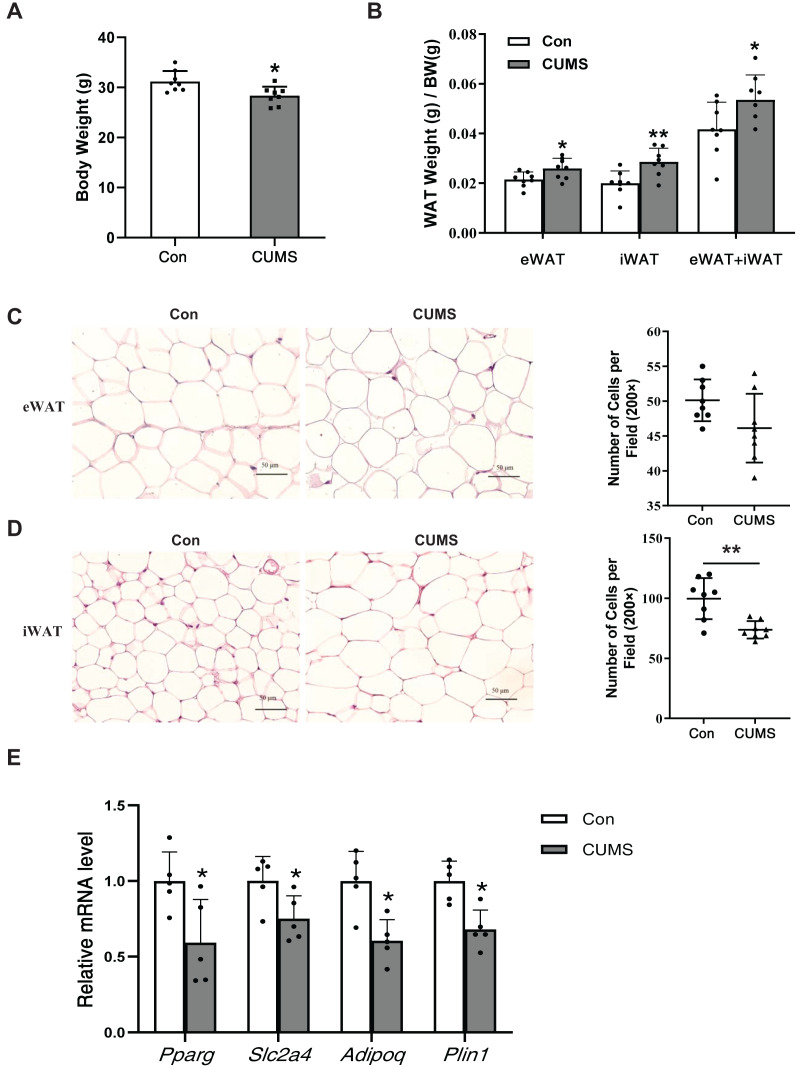
CUMS increases body fat percentage and enlarges iWAT adipocytes in WD ApoE^-/-^ mice. (A) Body weight decreased in the CUMS group after 12 weeks compared to the Con group (*n* = 8 per group); (B) ratio of adipose tissue to body weight in each group (the data are presented as mean ± SD; *p < 0.05, CUMS *vs* Con; *n* = 8 per group); (C and D) (Left) representative H&E staining images of adipocyte area in eWAT (C) and iWAT (D) in the Con and CUMS groups, 100x magnification; (Right) the number of adipocytes per field of view (the data are presented as mean ± SD; **p < 0.01, CUMS *vs* Con; *n* = 8 per group); and (E) relative *Pparg, Slc2a4, Adipoq*, and *Plin1* mRNA levels in iWAT in Con and CUMS groups (the data are presented as mean ± SD; *p < 0.05, CUMS *vs* Con; *n* = 5 per group).

CUMS has been depicted to reduce body weight in wild-type mice (Zhang et al., 2022). Body weight (BW), subcutaneous inguinal WAT (iWAT) weight, and visceral epididymis WAT (eWAT) weight were measured in WD ApoE^-/-^ mice. CUMS significantly decreased body weight while increasing the proportion of whole-body WAT mass (iWAT+eWAT) and the iWAT mass ratio in WD ApoE^-/-^ mice (Figs. 2A and 2B). The higher fat mass proportion in CUMS mice was most likely attributable to increased adipocyte size in iWAT (Figs. 2C and 2D). The downregulation of PPAR-γ, GLUT4, and other PPAR-γ targets is a common molecular feature of hypertrophic adipocytes (Aprile et al., 2020). CUMS significantly decreased *Pparg* and *Slc2a4* mRNA expression (Fig. 2E). Adiponectin is a direct target of PPAR-γ (Tontonoz & Spiegelman, 2008) and a well-known beneficial adipokine that attenuates vascular inflammation, increases fuel oxidation, and improves insulin sensitivity (Ghadge, Khaire & Kuvalekar, 2018). Perilipin 1 (PLIN1) is associated with the biogenesis of lipid droplets. Adipose tissue-specific expression of *Plin1* is transcriptionally regulated by PPAR-γ (Arimura et al., 2004). As expected, iWAT expression of *Adipoq* and *Plin1* mRNA was decreased in the CUMS group compared to the control group, as expected (Fig. 2E). However, CUMS only significantly decreased *Slc2a4* mRNA expression in eWAT (Fig. S1).

### CUMS increased lipid synthesis and decreased lipid hydrolysis in iWAT

**Figure 3:**
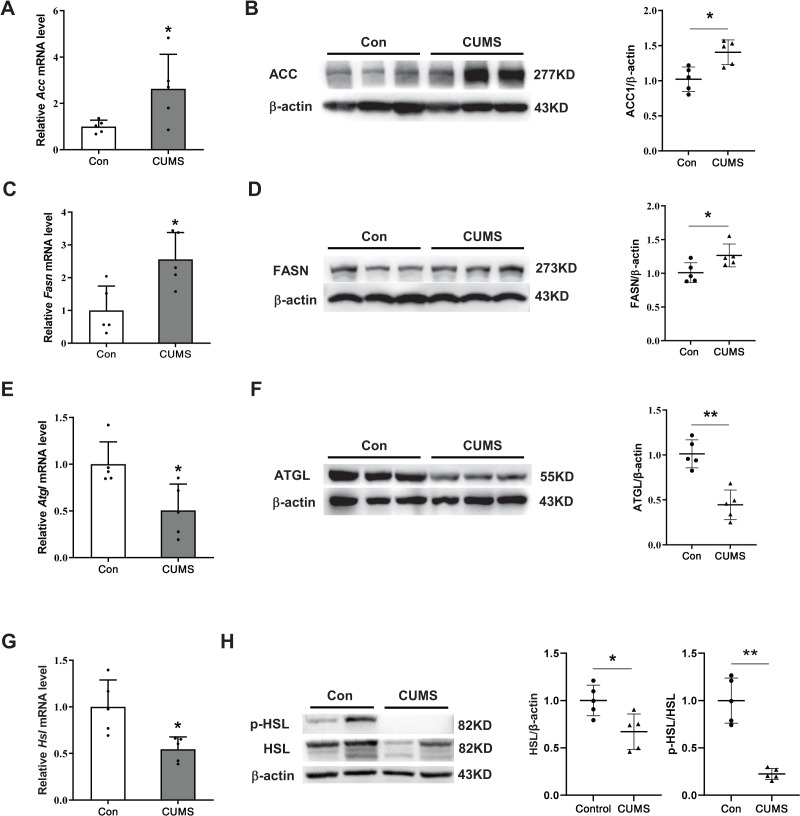
CUMS increases lipid synthesis and decreases lipid hydrolysis in iWAT. (A, C, E, and G) Relative mRNA levels of *Acc* (A), *Fasn* (C), *Atgl* (E), and *Hsl* (G) in iWAT (the data are presented as mean ± SD; *p < 0.05, **p < 0.01, CUMS *vs* Con; *n* = 5 per group); representative western blot and relative quantitative grayscale analysis of ACC (B), FASN (D), ATGL (F), HSL and p-HSL (H) expression in iWAT (the data are presented as mean ± SD; *p < 0.05, CUMS *vs* Con; *n* = 5 per group).

To elucidate the potential mechanism by which CUMS induces adipocyte hypertrophy, the study examined key enzymes involved in lipid synthesis and lipid hydrolysis in the iWAT. During the synthesis of fatty acids, the carboxylation of acetyl CoA into malonyl CoA is the first committed step, wherein ACC acts as the catalyst. FASN catalyzes the production of long-chain fatty acids by acetyl CoA and malonyl CoA (Cronan & Waldrop, 2002; Ortega et al., 2010). This research found that CUMS raised the expression of ACC and FASN at both the transcriptional and protein levels (Figs. 3A–3D). The initial step of triglyceride hydrolysis is catalyzed by the enzyme adipose triglyceride lipase (ATGL). In adipose tissue, hormone-sensitive lipase (HSL) converts triacylglycerols into fatty acids by hormone-regulated neutral lipase activity (Nielsen et al., 2014). The findings revealed that CUMS significantly inhibited ATGL, HSL, and phosphorylated hormone-sensitive lipase (p-HSL) expression in iWAT (Figs. 3E and 3H). These key enzymes of lipid metabolism were also detected in eWAT. However, CUMS did not significantly alter their mRNA levels (Fig. S2). The results suggest that increased lipid synthesis and decreased lipid hydrolysis may be responsible for the increase in the size of iWAT adipocytes.

### CUMS decreased ApoA-I expression in iWAT

**Figure 4:**
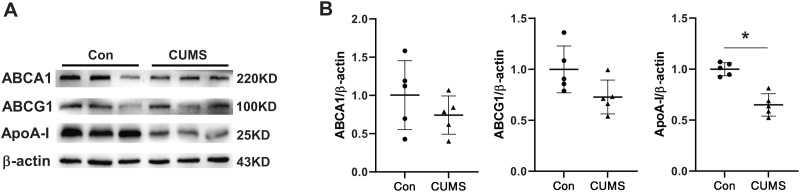
CUMS decreases ApoA-I expression in iWAT. (A) Representative western blot images of ABCA1, ABCG1, and ApoA-I expression in iWAT (*n* = 5 per group) and (B) relative quantitative grayscale analysis of ABCA1, ABCG1, and ApoA-I expression. The data are expressed as mean ± SD. *p < 0.05, CUMS *vs*. Con.

The aforementioned findings demonstrated that plasma HDL was downregulated in CUMS mice. Several epidemiological studies have demonstrated that HDL is a significant atherosclerosis risk factor (Pirillo, Casula & Olmastroni, 2021). Therefore, HDL-associated proteins in adipose tissue were examined. Hepatocytes or adipocytes are the primary cells that produce and secrete apolipoprotein A1 (ApoA-I) (Liao et al., 2018). ApoA-I, the most abundant protein in HDL, is well-known for its anti-atherogenic effects (Su & Peng, 2020b; Vázquez-Oliva et al., 2018). The ATP binding cassette transporter A1 (ABCA1) plays a crucial role in transporting excess adipocyte-free cholesterol to ApoA-I and other amphipathic apolipoproteins, leading to the formation of nascent HDL particles. Alternatively, cholesterol can also be transported to plasma HDL particles through the scavenger receptor class B type I (SR-BI) and ATP binding cassette transporter G1 (ABCG1) (Zhang et al., 2010). CUMS remarkably inhibited the expression of ApoA-I and tended to downregulate ABCA1 and ABCG1 (Fig. 4). The findings suggested that the decrease in plasma HDL level in CUMS mice may be related to ApoA-I down-regulation in adipose tissue. However, the exact mechanism requires further investigation. In addition, a recent study has found that ApoA-I knockout mice expressed ATGL and p-HSL less than WT mice, and the lipid metabolism genes were differentially expressed in WAT (Wei et al., 2014). These findings suggest that ApoA-I downregulation by CUMS may be one of the pathways that inhibit lipid hydrolysis and serves as a key link in adipose hypertrophy.

### CUMS increased mRNA levels of inflammatory cytokines in iWAT

**Figure 5:**
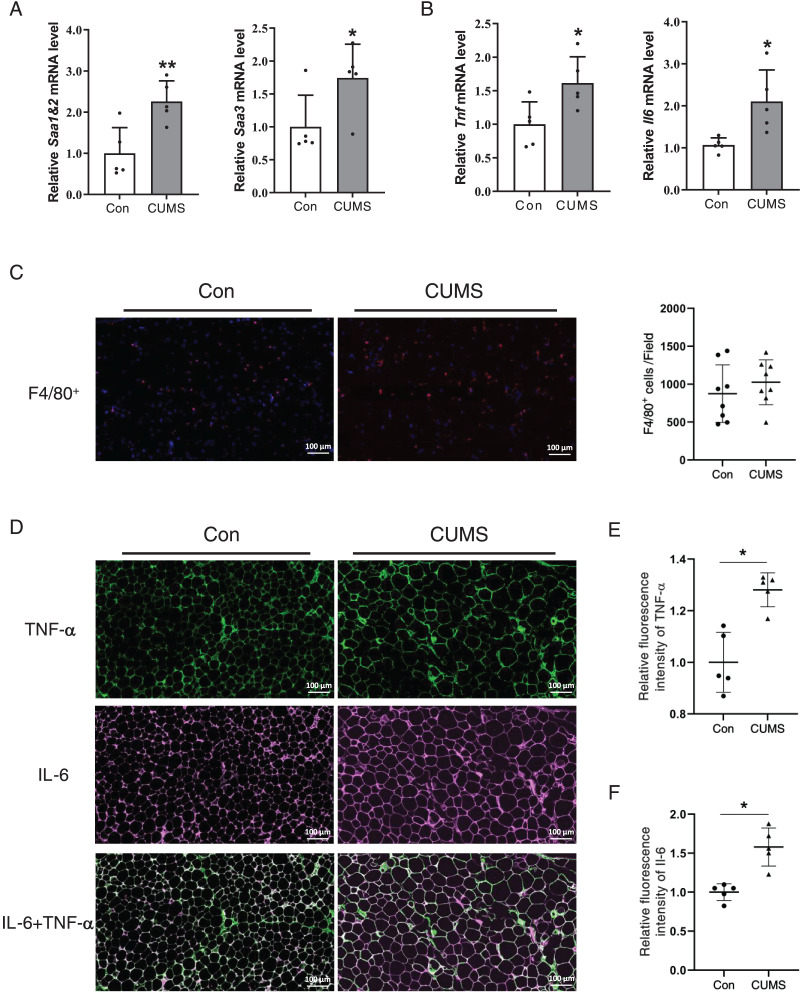
CUMS increases mRNA levels of inflammatory cytokine in iWAT. (A) Relative *Saa1/2* and *Saa3* mRNA levels in iWAT in Con and CUMS groups (*n* = 5/group); (B) relative *Tnf* and *Il6* mRNA levels in iWAT in Con and CUMS groups (the data are expressed as mean ± SD . *p < 0.05, **p < 0.01, CUMS *vs*. Con; *n* = 5 per group) ; (C) representative immunofluorescence images of macrophage marker F4/80 in iWAT inCon and CUMS groups, 200x magnification (Left) and quantification of F4/80-positive cells in each field of view (Right) (the data are presented as means ± SD, *n* = 8 per group); (D) representative immunofluorescence images of TNF-α (Top), IL-6 (Middle), and IL-6 + TNF-α (Bottom) in the Con and CUMS groups, 200x magnification; relative fluorescence intensity of TNF-α (E) and IL-6 (F) in the Con and CUMS groups (the data are presented as means ± SD. *p < 0.05, CUMS *vs*. Con; *n* = 5 per group).

Hypertrophied adipocytes exhibit modified metabolic properties, which include an increase in adipokine secretion, such as inflammatory cytokines. Serum amyloid A (SAA) is a newly discovered adipokine. Multiple studies have revealed that SAA accelerates the inflammatory process (Fasshauer et al., 2004), and influences the function of HDL (Sack, 2020). High levels of TNF-α and IL-6 are also characterized in hypertrophic WAT. Therefore, the inflammatory factors in adipose tissue were examined. The RNA analysis revealed that all these proinflammatory cytokines significantly increased in the CUMS group (Figs. 5A and 5B, Fig. S3). The results demonstrated that CUMS significantly increased the mRNA expression of all these inflammatory cytokines in iWAT. However, in eWAT, only Saa1 and Saa2 showed a significant increase in mRNA expression. In addition to adipocytes, macrophages play an important role in adipose tissue inflammation. The infiltration profile of macrophages was assessed using an immunofluorescence assay. Macrophages were labeled with the marker F4/80 (in red), while the sources of the inflammatory factors were monitored by labeling TNF-α (in green) and IL-6 (in pink). The findings revealed that CUMS did not increase macrophage infiltration of adipose tissue (Fig. 5C) and that adipocytes were the main source of TNF-α and IL-6 (Fig. 5D). Immunofluorescence semi-quantitative analysis also showed that CUMS significantly up-regulated the protein expression of inflammatory factors TNF-α and IL-6 in adipose tissue (Figs. 5E and 5F).

### CUMS inhibited the insulin pathway in iWAT and caused insulin resistance in mice

**Figure 6:**
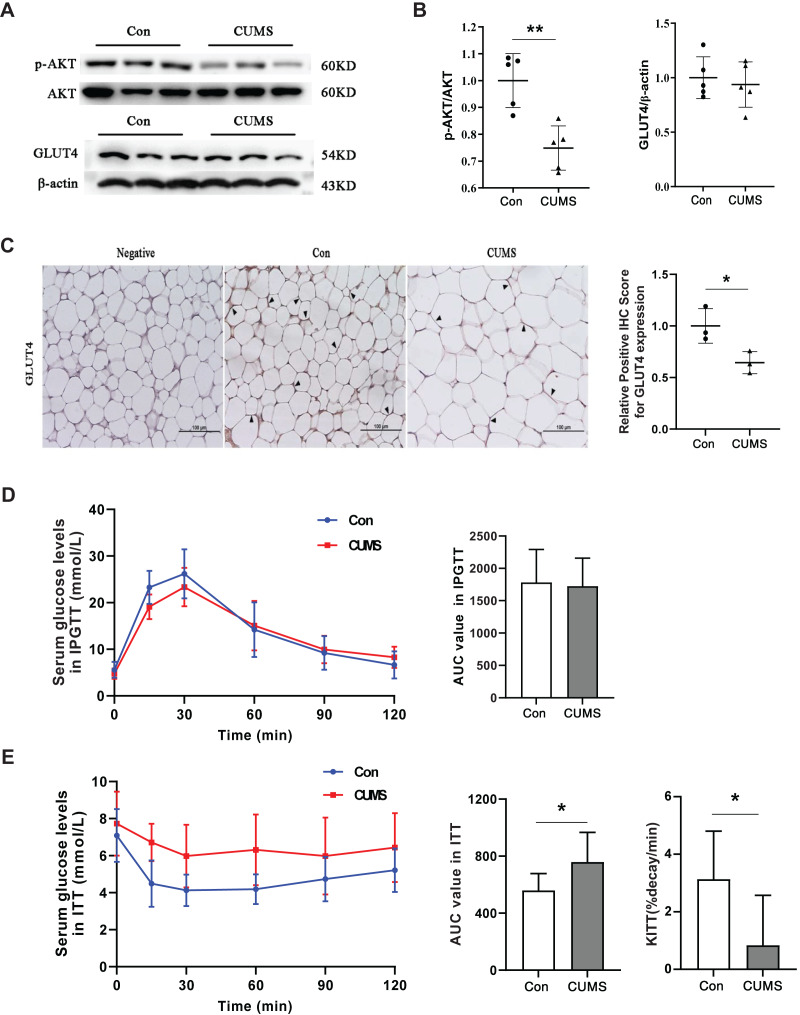
CUMS inhibits the insulin pathway in iWAT and causes insulin resistance in mice. (A) Representative western blot images of AKT, p-AKT, and GLUT4 expression in iWAT; (B) relative quantitative grayscale analysis of p-AKT/AKT (left) and GLUT4/β-actin (right) expression (the data presented as mean ± SD. **p < 0.01, CUMS *vs* Con; *n* = 5 per group); (C) (left) representative immunohistochemistry images of GLUT4 expression on the cell membrane of adipocytes in iWAT, the black arrows point to the positive area; (right) relative positive IHC score for GLUT4 expression (the data presented as mean ± SD. *p < 0.05, CUMS *vs* Con; *n* = 3 per group); (D) serum glucose levels (left) and AUC value (right) of serum glucose levels determined in IPGTT; (E) serum glucose levels (left) AUC value of serum glucose levels (middle) and the constant of plasma glucose disappearance (KITT) (right) determined in ITT *n* = 8/group. The data are presented as mean ± SD, *p < 0.05, CUMS *vs* Con.

Hypertrophic adipocytes contribute to insulin resistance in adipose tissue. Beyond muscles, adipose tissue is the most important effector of insulin. Adipocytes possess GLUT4 on their cell surface, which is responsible for transporting glucose from the extracellular milieu into the cell (Li et al., 2019). This transporter plays a crucial role in insulin resistance (Govers, 2014; Petersen & Shulman, 2018). AKT facilitates insulin-stimulated glucose entry in adipocytes by increasing GLUT4 trafficking (Khan & Kamal, 2019). Therefore, the AKT/GLUT4 pathway was investigated in this study. Western blot analysis demonstrated that CUMS significantly inhibited AKT phosphorylation without affecting GLUT4 expression (Figs. 6A and 6B). The immunohistochemical analysis revealed that CUMS led to a significant reduction in GLUT4 translocation to the cell surface, aligning with the decrease in AKT phosphorylation (Fig. 6C). Following CUMS, IPGTT, and ITT were performed. The glucose tolerance of the mice remained unaffected by CUMS, but insulin sensitivity was considerably decreased (Figs. 6D and 6E).

## Discussion

Metabolic abnormalities are more commonly linked to excess body fat rather than high lean body mass. Adipose tissue plays a pivotal role in regulating body homeostasis and influencing the development of inflammation or insulin resistance (Kawai, Autieri & Scalia, 2021). Although CUMS causes weight loss (Du Preez et al., 2021; Zhang et al., 2022) and osteosarcopenic adiposity (Ilich et al., 2020), its effect on adipocyte morphology and function is unknown. The findings revealed that CUMS increased WAT mass, particularly iWAT, in ApoE^−/−^ mice. This was accompanied by adipocyte hypertrophy, resulting in adipokine secretion disorder, insulin resistance, and decreased serum HDL levels, ultimately aggravating vascular atherosclerosis.

A recent study has found that CUMS promoted atherosclerosis in ApoE^-/-^ mice (Gu et al., 2019), but the mechanism needs to be investigated further. WAT serves as an energy reservoir, and its dysfunction is linked to atherosclerotic cardiovascular disease and dyslipidemia (Kershaw & Flier, 2004; Su & Peng, 2020a). Increasing evidence indicates that adipose tissue is involved in many metabolic activities, including cholesterol efflux and hormone secretion. Both plasma HDL cholesterol levels and overall reverse cholesterol transport may be affected by these activities. Changes in the morphology and function of adipose tissue influence plasma HDL levels and function (Zhang et al., 2019). The findings indicate that CUMS may decrease serum HDL levels *via* adipose tissue dysfunction. Proinflammatory cytokines downregulate ApoA-I expression in hepatocytes and animal plasma (Ryan, Taylor & McNicholas, 2009), suggesting that these cytokines may be independent predictors of atherosclerotic cardiovascular disease. Conversely, some adipokines, such as adiponectin, exert a protective effect against the development of atherosclerosis, but their levels decrease with increasing adiposity (Zhao, Kusminski & Scherer, 2021). The findings revealed that the levels of anti-atherogenic adipokines adiponectin and perilipin1 as well as the expression of HDL-related reverse transporters, particularly ApoA-I, were decreased in the adipose tissue of CUMS mice. In contrast, the levels of pro-atherogenic adipokines IL-6, TNF-α, and SAA were elevated. These results indicated that adipose tissue dysfunction may be responsible for the decrease in circulating HDL levels in CUMS mice. The exact signaling pathways require further investigation.

Triacylglycerol (TG) is primarily stored in white adipocytes as lipid droplets, and the balance of white adipocyte metabolism depends on the proper regulation of lipogenesis and lipolysis (Morigny et al., 2021). ACC and FASN are vital enzymes involved in *de novo* lipogenesis, forming a significant component of this process, whereas HSL and ATGL serve as key enzymes involved in mediating lipolysis. In this study, the effects of CUMS on these key enzymes were observed at the transcriptional and protein levels. While FASN is primarily regulated at the transcriptional level, ACC activity undergoes regulation through processes such as phosphorylation, protein conformation, and polymerization. However, the intricate mechanisms behind these regulatory processes necessitate further investigation (Wilhelm, Rossmeislova & Siklova, 2022). In the subcutaneous adipose tissue of obese individuals, the mRNA and protein expression levels of ATGL and HSL have previously been contradictorily reported (Nielsen et al., 2014). Several studies have found that HSL protein levels are lowered in obese individuals (Lafontan & Langin, 2009; Nielsen et al., 2014; Ray et al., 2009). FFA, which is the product of lipolysis, is known to have consistently elevated levels in obese patients (Lafontan & Langin, 2009; Morigny et al., 2021). In this study, the plasma FFA levels did not vary considerably between the two groups. Interestingly, the iWAT of CUMS mice exhibited reduced levels of ATGL and HSL in both mRNA and protein expression, with a substantial suppression of phosphorylated HSL. This suggests a decreased stimulation of adipose tissue lipolysis in ApoA-I^-/-^ mice, as evidenced by the lower levels of p-HSL and lipolytic enzyme mRNA. Consequently, the ApoA-I^-/-^ mice gained significantly more body fat compared to their WT counterparts. In contrast with ApoA-I^-/-^ mice, ApoA-I^tg/tg^ mice weighed less than WT mice and exhibited higher levels of p-HSL and lipolytic enzyme mRNA in their adipose tissue depicting elevated lipolysis of the adipose tissue (Wei et al., 2014). Therefore, based on these experimental results, it was speculated that the remarkable decrease in p-HSL expression level may be related to the reduced ApoA-I expression level in iWAT. The results indicated that the pattern of adipocyte hypertrophy induced by CUMS might differ from the typical state of obesity. The key mechanism underlying this difference appears to be the inhibition of HDL-associated protein expression.

Insulin catalyzes glucose uptake into skeletal muscle and adipose tissue *via* activating the phosphatidylinositol-3-kinase/Akt signaling pathway and subsequent translocation of GLUT4 from intracellular storage vesicles to the plasma membrane (Li et al., 2019). The results showed that CUMS inhibited the phosphorylation of AKT and decreased the expression of GLUT4 on the adipocyte surface, suggesting that CUMS impaired the insulin signal and glucose transport.

Since changes in subcutaneous WAT were more significant than changes in visceral WAT in CUMS mice, this study focused on changes in subcutaneous WAT. Nevertheless, it is crucial to further investigate the changes in visceral WAT since it is recognized as an independent risk factor for cardiovascular and metabolic morbidity and mortality (Neeland et al., 2019). This research is the first comprehensive investigation of the effects of CUMS on the body’s adipose tissue, aiming to elucidate the principles and pathways of atherosclerosis aggravation caused by CUMS from the perspective of adipose tissue dysfunction.

## Conclusions

These findings indicate that CUMS induces dysfunction in adipose tissue *via* a mechanism that results in dyslipidemia, insulin resistance, and increased inflammation. The process may be aided by the decreased expression of HDL-associated proteins in adipose tissue. These findings identify a novel mechanism *via* which CUMS aggravates atherosclerosis and may suggest a potential therapeutic target for this disease.

### Sample size

According to previous work, the ratio of lesion area per lumen area was used to evaluate the severity of atherosclerosis (Zhang et al., 2022). Therefore this indicator was applied to determine sample size in atherosclerosis hypothesis testing. The mean ratio of lesion area per lumen area of the control group was calculated to be 0.3039 ± 0.1732, and the lesion area per lumen area of the expected stress group increased by 30%, establishing a bilateral alpha of 0.05 and a confidence interval of 90%. According to the sample size calculation formula, it was determined that the control and experimental groups would require eight mice each.

### Study limitations

The present study applied atherosclerosis model mice (ApoE^-/-^) as the study subject, which may lead to species limitations and gene knockout limitations.

### Statement

The protocol was prepared before the study and was not registered.

## Supplemental Information

10.7717/peerj.16029/supp-1 Supplemental Information 1 Relative Pparg, Slc2a4, Adipoq, and Plin1 mRNA levels in eWAT in Con and CUMS groups. The data are presented as mean ± SD; *p < 0.05, CUMS *vs* Con; *n* = 5 per group.

10.7717/peerj.16029/supp-2 Supplemental Information 2 Relative Acc, Fas, Atgl, and Hsl mRNA levels in eWAT in Con and CUMS groups. The data are presented as mean ± SD; *p < 0.05, CUMS *vs* Con; *n* = 5 per group.

10.7717/peerj.16029/supp-3 Supplemental Information 3 Relative Acc, Fas, Atgl, and Hsl mRNA levels in eWAT in Con and CUMS groups. The data are presented as mean ± SD; *p < 0.05, CUMS *vs* Con; *n* = 5 per group.

10.7717/peerj.16029/supp-4 Supplemental Information 4 Raw data for the figures.

10.7717/peerj.16029/supp-5 Supplemental Information 5 WB Raw Data. All groups and samples are circled with red boxes and labeled with specific information in the figures.

10.7717/peerj.16029/supp-6 Supplemental Information 6 The ARRIVE guidelines 2.0: author checklist.

## Additional Information and Declarations

### Data Availability

The following information was supplied regarding data availability: The raw data are available in the Supplemental File.
