# Supplementary material for: Chronic unpredictable mild stress promotes atherosclerosis via adipose tissue dysfunction in ApoE-/- mice
Source: PeerJ. 2023 Sep 4;11:e16029. doi: 10.7717/peerj.16029 (PMC10484201; doi:10.7717/peerj.16029)
Supplement: Supplemental Information 2 — The data are presented as mean ± SD; *p < 0.05, CUMS vs Con; n = 5 per group. [file peerj-11-16029-s002.pdf]

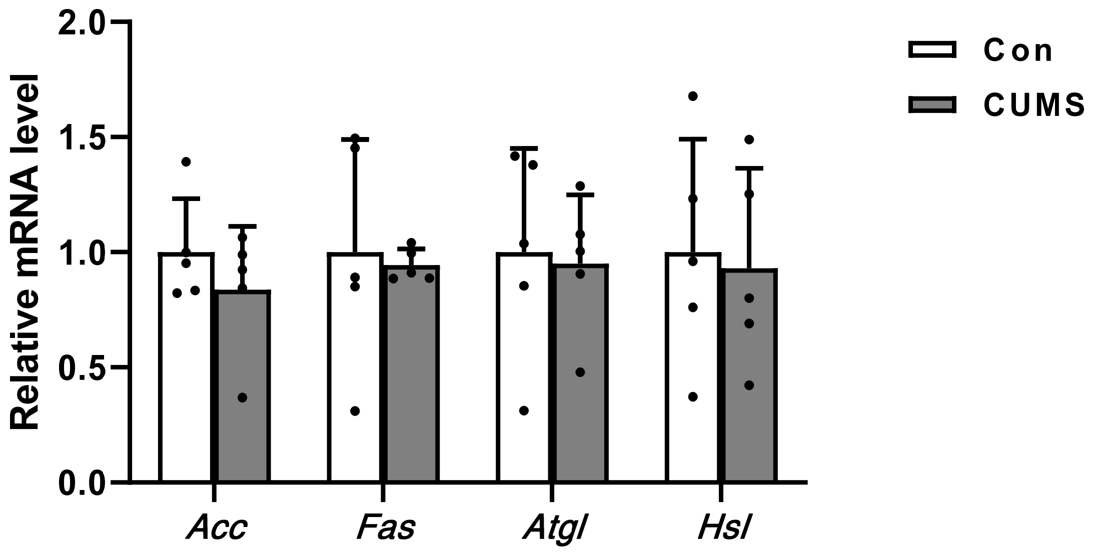

Supplemental Figure 2. Relative *Acc* , *Fas*, *Atgl*, and *Hsl* mRNA levels in eWAT in Con and CUMS groups. The data are presented as mean ± SD; *$p < 0.05$, CUMS vs. Con; n = 5 per group.
