# Supplementary material for: Chronic unpredictable mild stress promotes atherosclerosis via adipose tissue dysfunction in ApoE-/- mice
Source: PeerJ. 2023 Sep 4;11:e16029. doi: 10.7717/peerj.16029 (PMC10484201; doi:10.7717/peerj.16029)
Supplement: Supplemental Information 5 — All groups and samples are circled with red boxes and labeled with specific information in the figures. [file peerj-11-16029-s005.pdf]

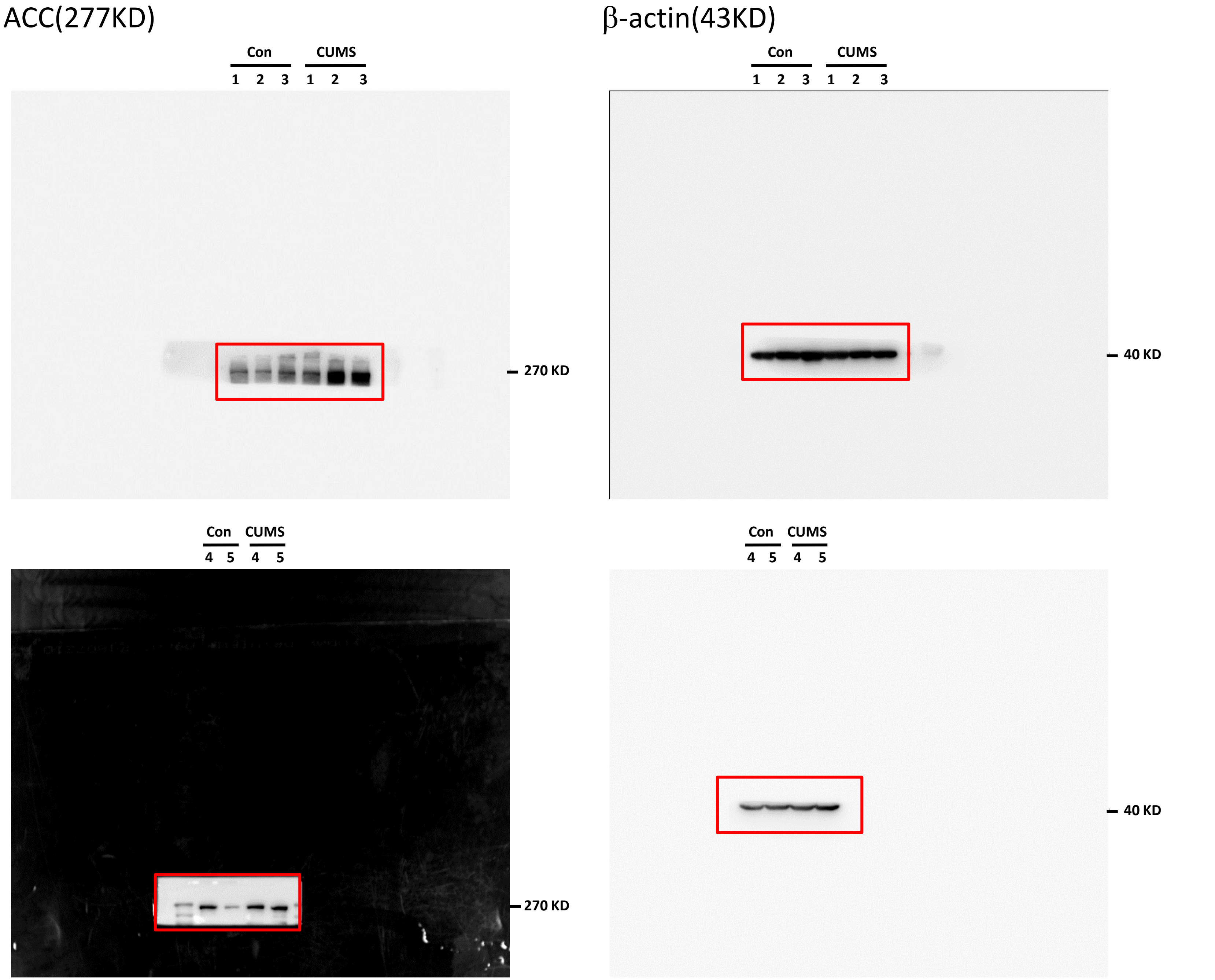
ACC(277KD)
β-actin(43KD)
Con
CUMS
1 2 3 1 2 3
270 KD
40 KD
Con
CUMS
4 5 4 5
270 KD
40 KD

FAS(273KD)

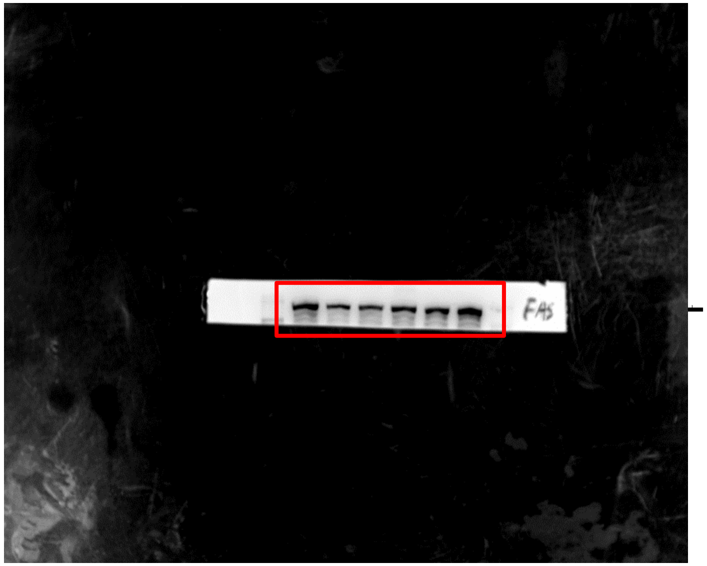

β-actin(43KD)

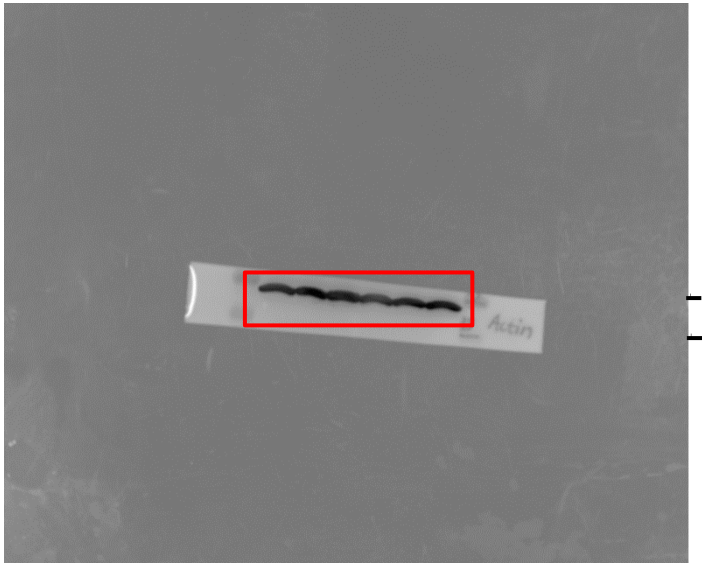

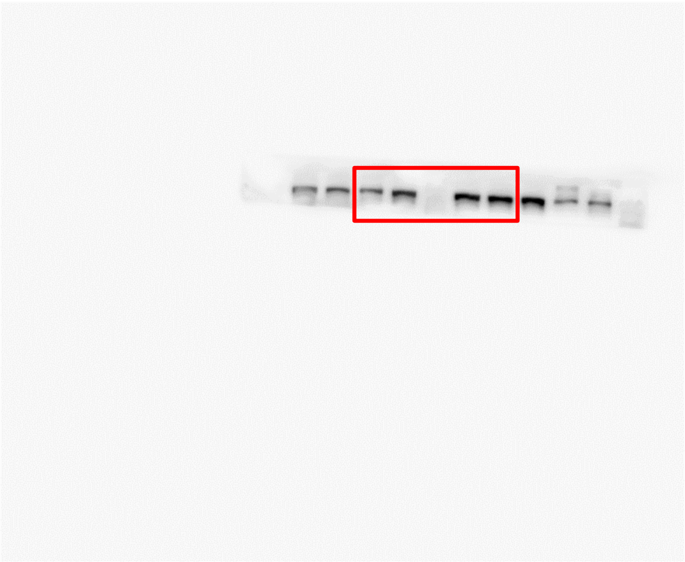

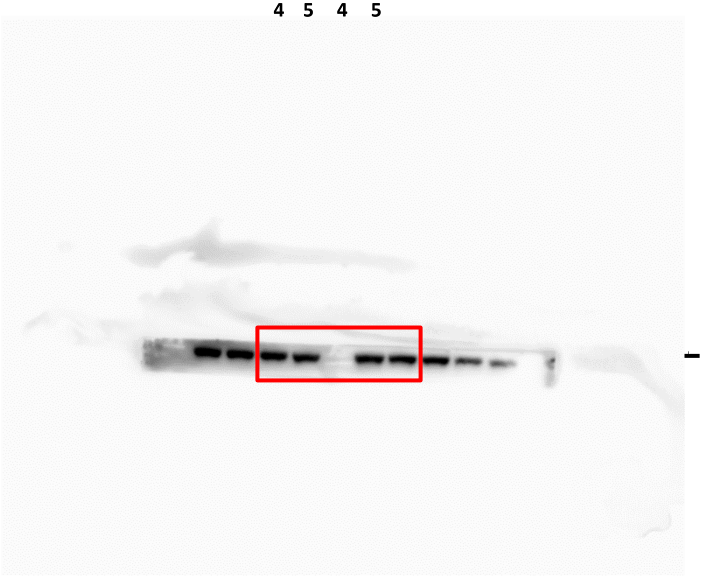

ATGL(55KD)

β-actin(43KD)

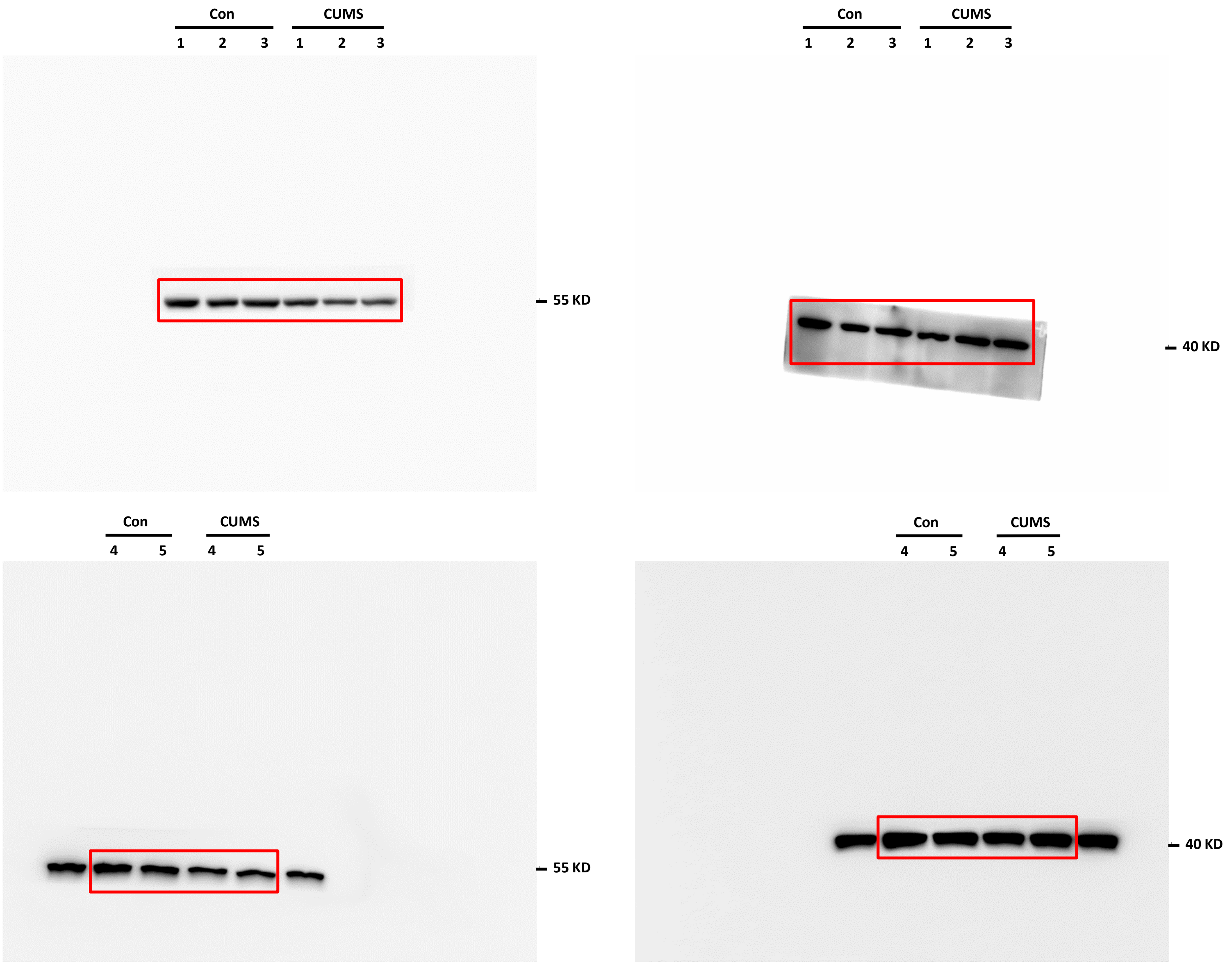

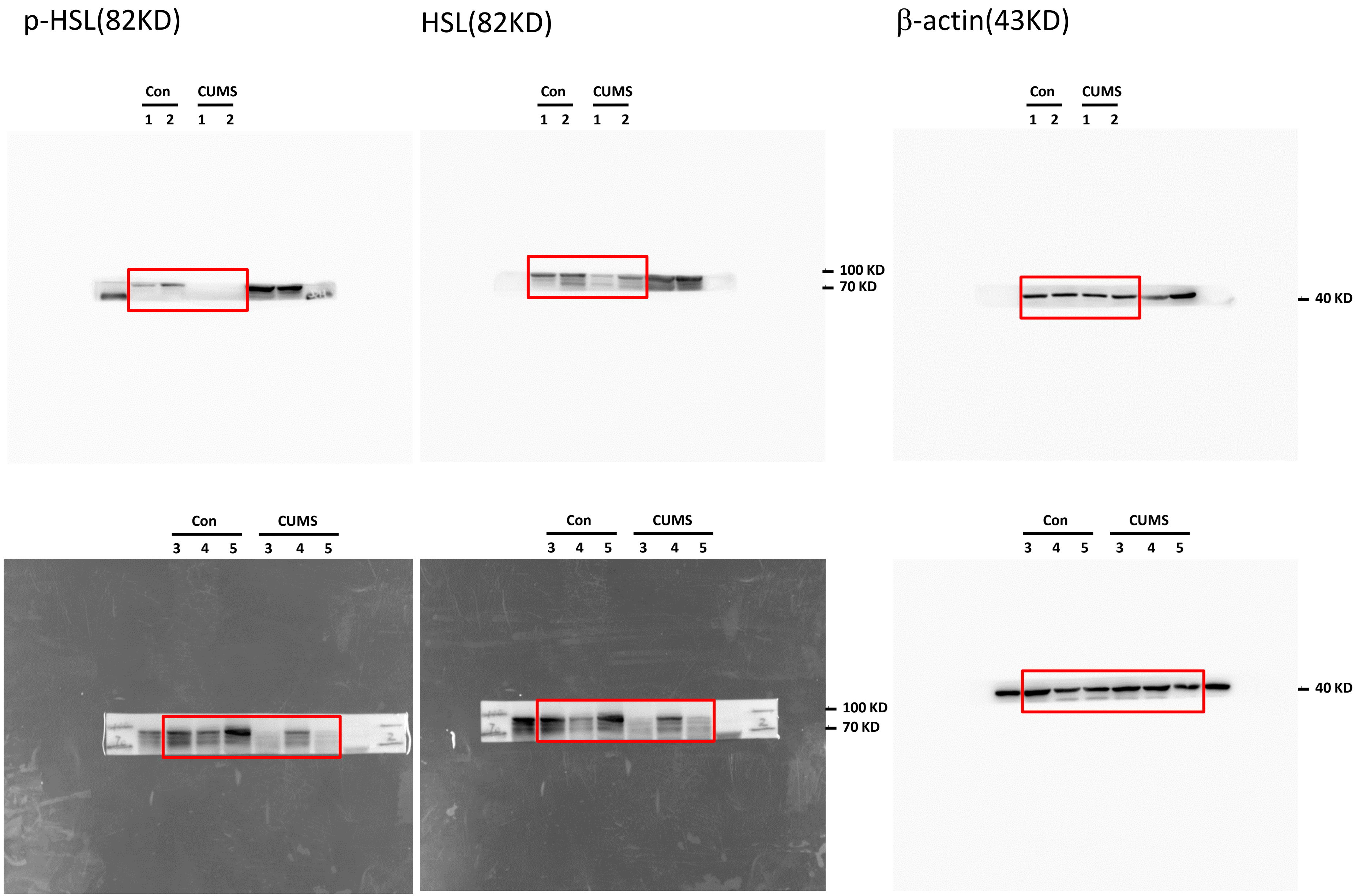

p-HSL(82KD)
HSL(82KD)
β-actin(43KD)
Con
CUMS
1 2 1 2
100 KD
70 KD
40 KD
Con
CUMS
3 4 5 3 4 5

p-AKT(60KD)

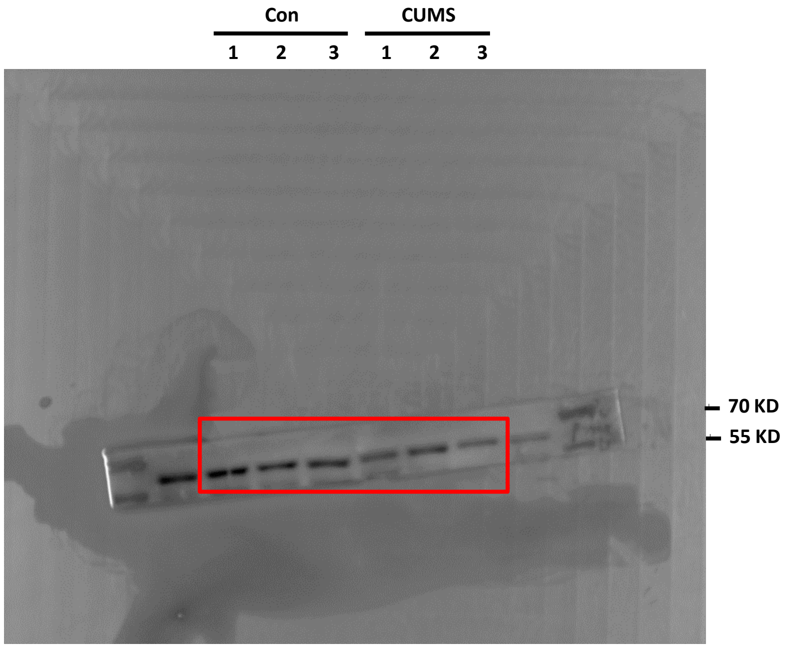

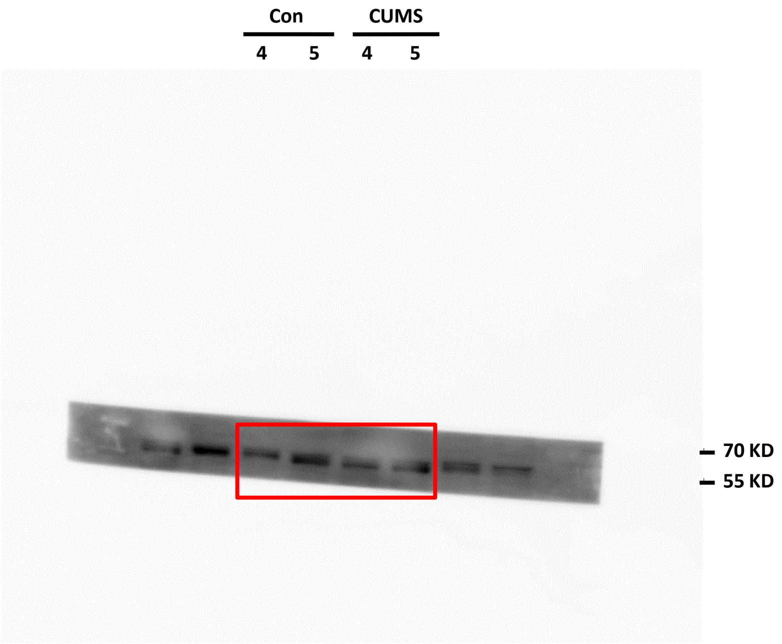

AKT(60KD)

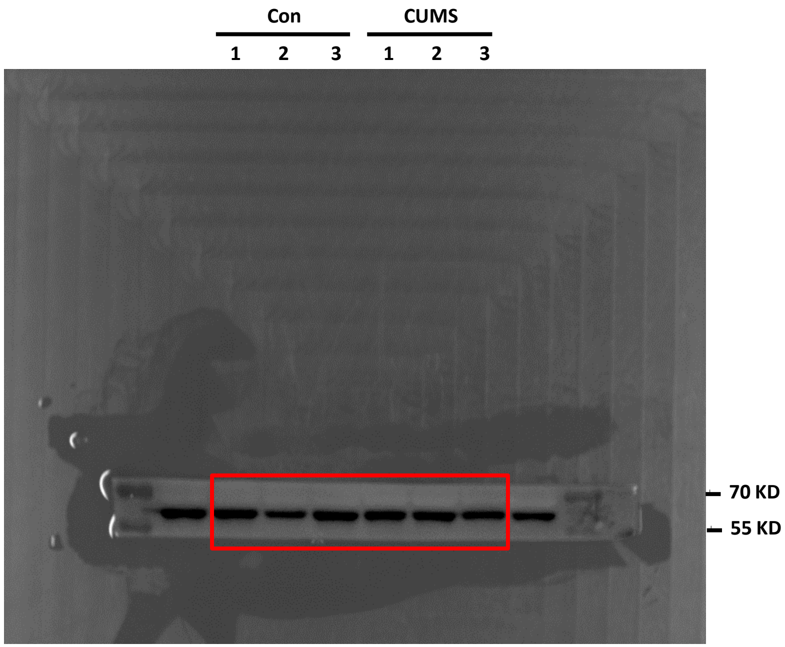

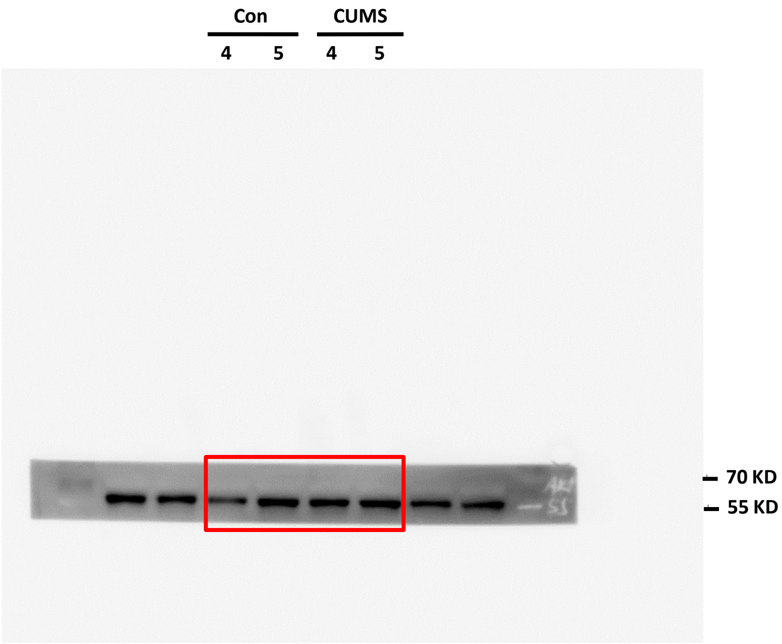

GLUT4(54KD)

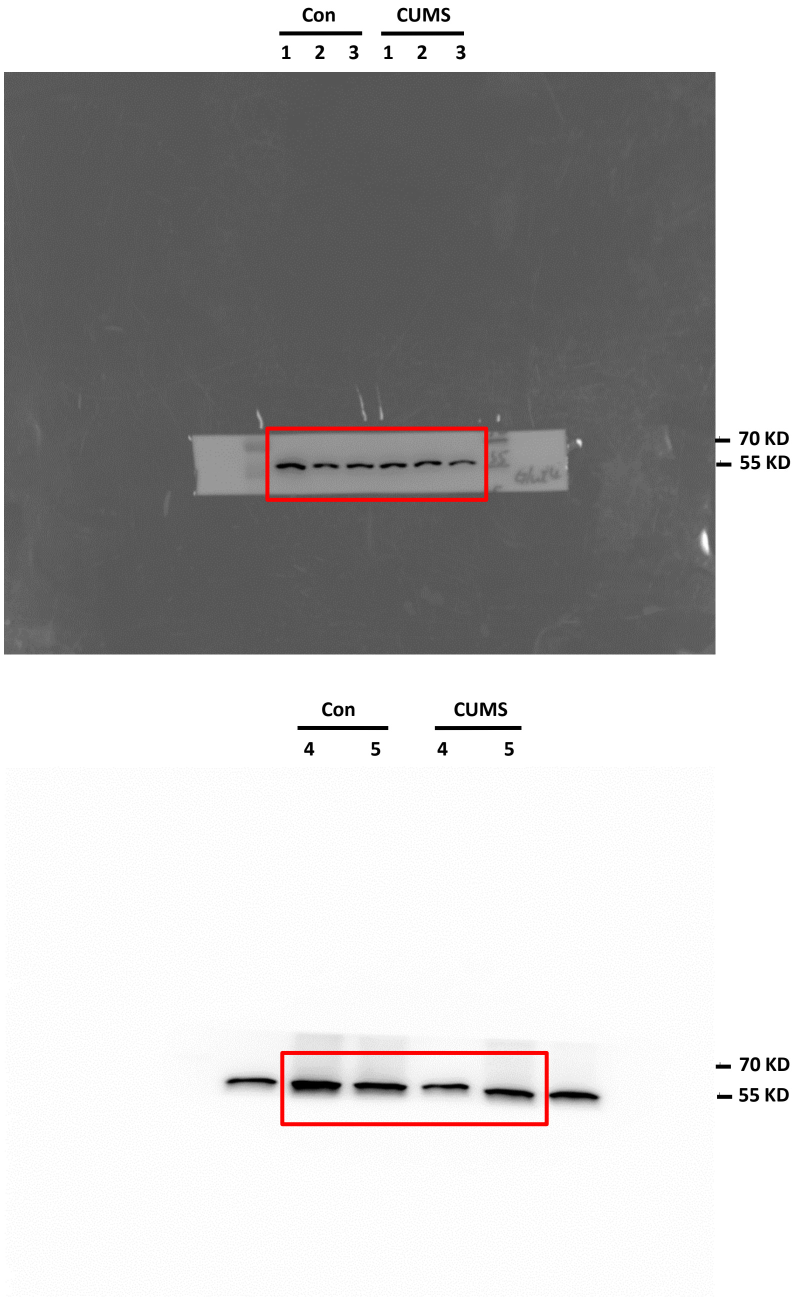

β-actin(43KD)

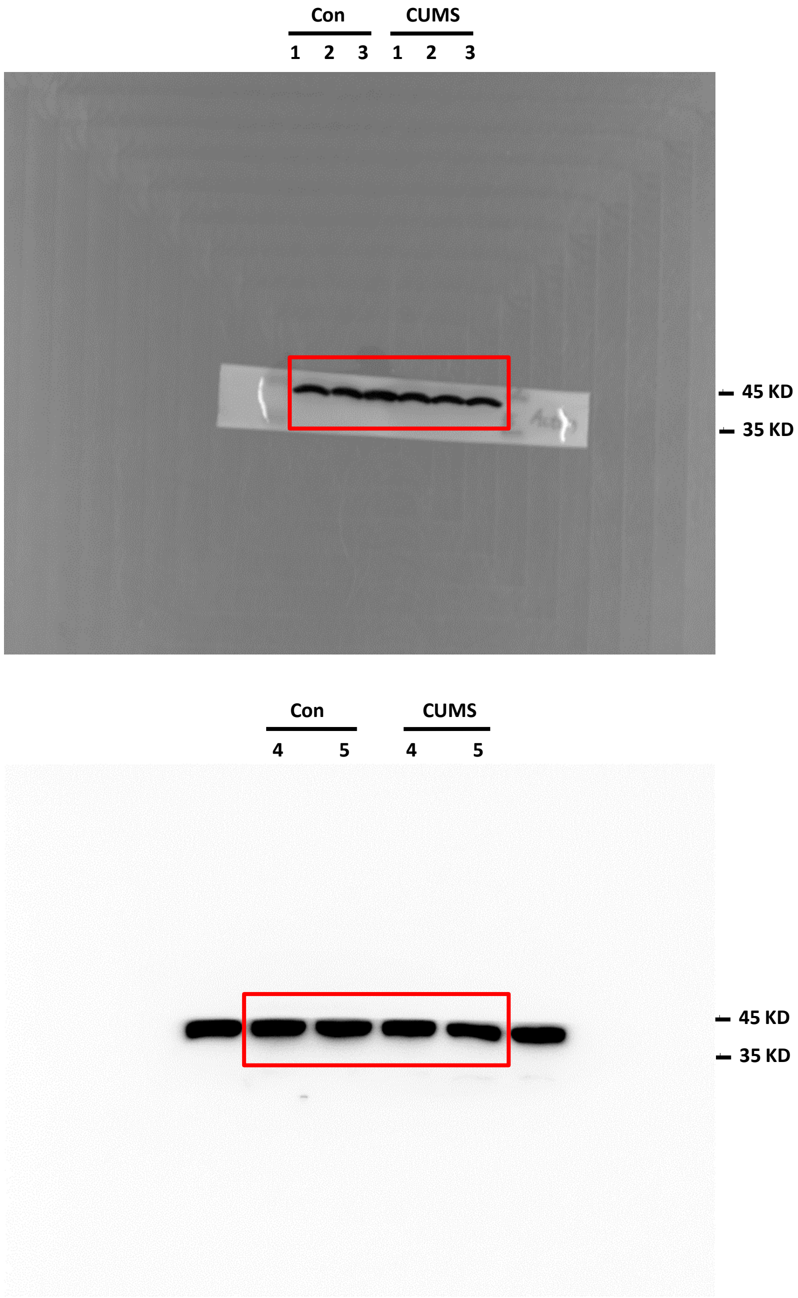

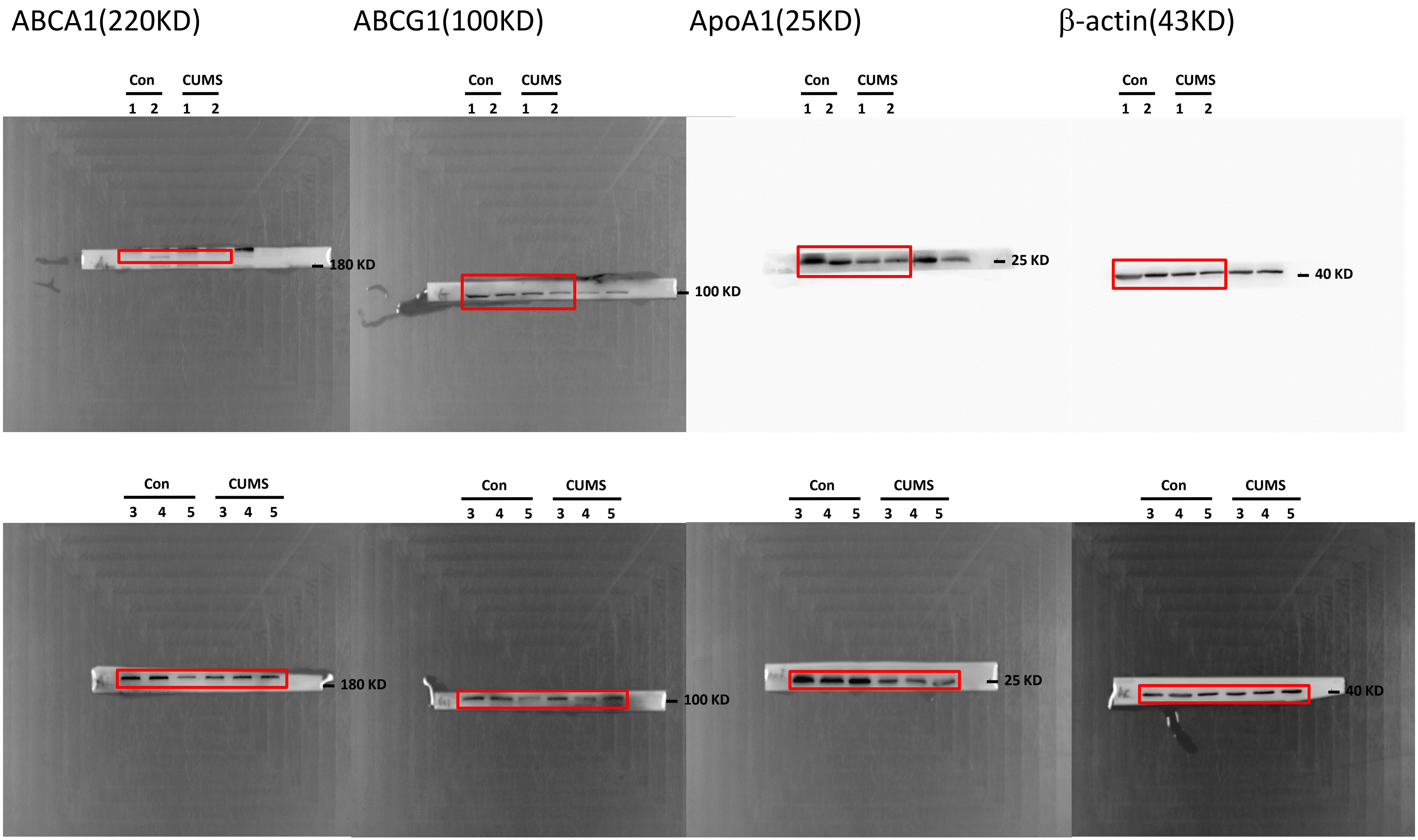
ABCA1(220KD)
ABCG1(100KD)
ApoA1(25KD)
β-actin(43KD)
Con
CUMS
1 2 1 2
180 KD
100 KD
25 KD
40 KD
3 4 5 3 4 5
